# Habitual Tea Consumption and Risk of Fracture in 0.5 Million Chinese Adults: A Prospective Cohort Study

**DOI:** 10.3390/nu10111633

**Published:** 2018-11-02

**Authors:** Qian Shen, Canqing Yu, Yu Guo, Zheng Bian, Nanbo Zhu, Ling Yang, Yiping Chen, Guojin Luo, Jianguo Li, Yulu Qin, Junshi Chen, Zhengming Chen, Jun Lv, Liming Li, on behalf of the China Kadoorie Biobank Collaborative Group

**Affiliations:** 1 Department of Epidemiology and Biostatistics, School of Public Health, Peking University Health Science Center, Beijing 100191, China; qianshen@bjmu.edu.cn (Q.S.); yucanqing@pku.edu.cn (C.Y.); znb@pku.edu.cn (N.Z.); lmLee@vip.163.com (L.L.); 2 Chinese Academy of Medical Sciences, Beijing 100191, China; guoyu@kscdc.net (Y.G.); bianzheng@kscdc.net (Z.B.); 3 Clinical Trial Service Unit & Epidemiological Studies Unit (CTSU), Nuffield Department of Population Health, University of Oxford, Oxford OX1 2JD, UK; ling.yang@ndph.ox.ac.uk (L.Y.); yiping.chen@ndph.ox.ac.uk (Y.C.); zhengming.chen@ndph.ox.ac.uk (Z.C.); 4 Pengzhou Center for Disease Control and Prevention, Pengzhou 611930, Sichuan, China; L630415@163.com (G.L.); fintear@163.com (J.L.); 5 NCDs Prevention and Control Department, Liuzhou Center for Disease Control and Prevention, Liuzhou 545007, Guangxi, China; lzcdcmbfzk@126.com; 6 China National Center for Food Safety Risk Assessment, Beijing 100191, China; chenjunshi@cfsa.net.cn; 7 Key Laboratory of Molecular Cardiovascular Sciences (Peking University), Ministry of Education, Beijing 100191, China; 8 Peking University Institute of Environmental Medicine, Beijing 100191, China

**Keywords:** tea consumption, fracture, cohort study

## Abstract

Background: Tea consumption may have favorable effects on risk of fracture. However, little is known about such association in Chinese adults. The aim of this study was to examine the association between tea consumption and risk of hospitalized fracture in Chinese adults. Methods: The present study included 453,625 participants from the China Kadoorie Biobank (CKB). Tea consumption was self-reported at baseline. Hospitalized fractures were ascertained through linkage with local health insurance claim databases. The results: During a median of 10.1 years of follow-up, we documented 12,130 cases of first-time any fracture hospitalizations, including 1376 cases of hip fracture. Compared with never tea consumers, daily tea consumption was associated with lower risk of any fracture (hazard ratio (HR): 0.88; 95% confidence interval (CI): 0.83, 0.93). Statistically significant reduced risk of hip fracture was shown among daily consumers who most commonly drank green tea (HR: 0.80; 95% CI: 0.65, 0.97) and those who had drunk tea for more than 30 years (HR: 0.68; 95% CI: 0.52, 0.87). Our conclusions: Habitual tea consumption was associated with moderately decreased risk of any fracture hospitalizations. Participants with decades of tea consumption and those who preferred green tea were also associated with lower risk of hip fracture.

## 1. Introduction

Bone fractures usually result from the combination of impaired bone strength and trauma from falling [1,2]. Bone fractures may lead to reduced activities, functional impairment, disability, and even increased mortality of patients [3,4]. Furthermore, bone fractures are associated with enormous economic expenditure. Hip fractures account for the majority of this burden mainly because of their severity, and requirements of medical care and hospital facilities [4,5].

Tea is among the most widely consumed beverages in the world and rich in both caffeine and polyphenols. Experimental studies demonstrated that caffeine, in a high amount, may promote differentiation of osteoclast [6,7] and increase urinary calcium [8], leading to diminished bone mineral density. Polyphenols, however, were shown to have favorable effects on bone biology [9,10,11]. Previous epidemiological studies have yielded inconsistent associations between tea consumption and risk of fracture. Tea consumption was associated with a decreased risk of fracture in some case-control studies [12,13,14], but not in others [15,16,17]. Most of the prospective cohort studies observed null associations between tea consumption and risk of fracture [18,19,20,21,22], while one study reported a positive association [23] and another reported a negative one [24]. These prospective studies were primarily conducted in Western postmenopausal women, had small sample sizes with few fracture cases, and had a relatively low consumption of tea. Even the existing meta-analyses assessing the association between tea consumption and fracture were inconsistent. Chen et al. [25], Yan et al. [26], and Guo et al. [27] reported no association between tea consumption and fracture, whereas Sheng et al. [28] reported that individuals drinking 1–4 cups of tea per day was associated with a lower risk of hip fracture. Also, the associations with various measures of tea consumption, like the amount of tea leaves added, types of tea, and duration of tea consumption, have yet to be examined. For Chinese populations, in which tea is most widely consumed, only one case-control study [14] and one cohort study [22] were performed and the results were mixed.

Thus, we aimed to prospectively examine the association between tea consumption and risk of hospitalized fracture in approximately 0.5 million Chinese adults from the China Kadoorie Biobank (CKB).

## 2. Materials and Methods

### 2.1. Study Population

Details of the CKB study design and characteristics of the study participants have been described elsewhere [29,30]. Briefly, the baseline survey took place between 2004–2008 in 10 geographically diverse areas of China (five urban and five rural areas). In each area, all nondisabled, permanent residents aged 35–74 years were invited to participate. Overall, a total of 512,891 individuals were recruited, including a few just outside the age range of 35–74 years. Each participant completed an interview-administered questionnaire and physical measurements.

After correction for errors in age and exclusion of participants with age outside of the 30–79 years, 512,715 participants were eligible for inclusion in the study. In the present analysis, participants with a history of any fracture before baseline (*n* = 35,444) were excluded. We also excluded participants with other diseases known to affect fracture risk, including a self-reported doctor diagnosis of cancer (*n* = 2578), heart disease (*n* = 15,472), or stroke (*n* = 8884) at baseline [31]. We also excluded participants who were lost to follow-up shortly after baseline (*n* = 1), and whose information on body mass index (BMI) was missing (*n* = 2). The final analysis included 453,625 participants, including 181,566 men and 272,059 women.

Ethical approval was obtained from the Ethical Review Committee of the Chinese Centre for Disease Control and Prevention, Beijing, China, and the Oxford Tropical Research Ethics Committee, University of Oxford, UK. All study participants provided written informed consent.

### 2.2. Assessment of Tea Consumption

At baseline survey, we asked participants “during the past 12 months, how often did you drink any tea (never, only occasionally, only at certain seasons, every month but less than weekly, or at least once a week)?” Participants who drank tea at least once a week were further asked to report: (1) days drinking in a typical week (1–2 days, 3–5 days, or almost every day), (2) cups (in 300 mL-sized) of tea consumed on days of drinking, (3) times of changing tea leaves on days of drinking, (4) amount of tea leaves (in grams) added each time, (5) types of tea most commonly consumed (green tea, oolong tea, black tea, or others), and (6) age they started drinking tea weekly. A pictorial guide was provided to demonstrate the standard sized cup and the amount of tea leaves. The total tea leaves consumed (in grams) on days of drinking was calculated as the product of the amount of tea leaves added each time and the times of changing tea leaves. Duration of tea consumption was calculated as the difference between age at baseline and age of starting drinking tea weekly. According to tea consumption frequency, participants were categorized into three groups (never, less than daily, or daily consumers). Daily consumers were further categorized into four groups according to rounded quartiles of the amount of tea consumed in grams (0.1–2.0, 2.1–3.0, 3.1–5.0, or >5.0 g per day) or in cups (1–2, 3–4, 5–6, or ≥7 cups per day).

### 2.3. Assessment of Outcomes

Hospitalized fracture outcomes were ascertained periodically through linkage with local health insurance (HI) claim databases. The reimbursement data of the local HI data are comprehensive and capture information on all diagnoses and treatments prescribed to patients who sought health care in a hospital. Such linkage has been achieved for about 98% of our participants, which was similar across ten survey sites. Linkage to local HI databases was renewed annually. Participants who failed to be linked to local HI databases were actively followed annually by staff to ascertain their status including hospital admission, death, and moving out of the study area. Fractures that did not require hospital admission as an in-patient were not ascertained in the present study. The date of fracture incidence was based on the admission date from the hospital inpatient discharge summaries. Trained staff who were blind to participants’ baseline information coded the cause of incident cases with the 10th revision of the International Classification of Diseases (ICD-10). The present study focused on the first-time hospitalizations of interest during the follow-up. Hip fracture cases were defined with ICD-10 codes of S72.0, S72.1, and S72.2. Any fracture cases were defined with the codes of S12, S22, S32, S42, S52, S62, S72, S82, and S92. Fractures of other parts of neck (S12.8), flail chest (S22.5), scapula (S42.1), fingers (S62.5–62.7), and toes (S92.4, S92.5) as any fracture events were excluded since these fractures were less likely associated with osteoporosis [32].

### 2.4. Assessment of Covariates

Information on socio-demographic characteristics (age, sex, education, occupation, marital status, and household income), lifestyle (smoking, alcohol drinking, physical activity, and diet), self-reported medical history, and reproductive history (in women) was obtained from the baseline questionnaire. Daily level of physical activity was calculated by multiplying the metabolic equivalent tasks (METs) value for a particular type of physical activity by hours spent on that activity per day and summing the MET-hours for all activities. Habitual dietary intake in the past year was assessed by a qualitative food frequency questionnaire (FFQ). We conducted a reliability and validity study of the FFQ during 2015–2016. The study included 432 CKB participants who completed two FFQ (median interval: 3.3 months) and twelve 24-h dietary recalls (24-HDR). The reliability of the FFQ was assessed by comparing the frequency of food consumption from the two FFQ. Except for fresh vegetables, values of weighted kappa ranged from 0·62 to 0·90 for all food categories. As for fresh vegetables, exact agreement rate was 89.8%, and misclassification to opposite quartiles was less than 1.0%. The validity of the FFQ was evaluated by comparing the frequency of food consumption from the first FFQ and the multiple 24-HDR. Except for fresh vegetables, values of weighted kappa ranged from 0.60 to 0.90 for all food categories. As for fresh vegetables, the exact agreement rate was 89.3%, and misclassification to opposite quartiles was less than 0.3%. Height, weight, waist and hip circumferences, and blood pressure were obtained from physical measurements using standard protocol and validated instruments.

### 2.5. Statistical Analyses

Each participant’s follow-up time was accrued from baseline survey until the admission date of the first-time fracture hospitalization, death, loss to follow up, or 31 December 2016, whichever occurred first. Stratified Cox proportional hazards models, with age as the underlying time scale, were conducted to calculate hazard ratios (HRs) and 95% confidence intervals (CIs) between tea consumption and risk of fracture. Multivariable-adjusted models were stratified jointly by 10 study areas and age at baseline in 5-year intervals, and they were adjusted for sex, education, marital status, alcohol consumption, smoking status, physical activity, frequencies of red meat, fruit, vegetable, and dairy product consumption, menopause status (only for women), BMI, waist-to-hip ratio, prevalent hypertension, and diabetes. Tests for linear trend were only conducted in daily tea consumers by assigning the median value of tea consumption (in grams or cups per day) to each of the categories as a continuous variable in regression models. Also, we examined associations between tea consumption and risk of fracture according to types of tea and duration of tea consumption. Subgroup analyses were conducted to test for interaction of tea consumption with 11 baseline factors (for example, age, region, etc.) by using likelihood ratio tests comparing models with and without a cross-product term.

We used Stata, version 15.0 (StataCorp, College Station, TX, USA), for statistical analyses. All *p* values were two-sided. Statistical significance was defined as *p* < 0.05, except that a Bonferroni corrected *p* value (0.05/11) was used in the interaction analyses since multiple testing issues might occur.

## 3. Results

Of 453,625 participants, the mean age was 51.4 ± 10.6 years. Overall, 26.2% of participants drank tea daily, in which 41.3% of men and 16.1% of women were daily tea consumers. Table 1 summarizes baseline characteristics of the study participants according to tea consumption. Compared with never tea consumers during the past 12 months, participants who drank tea daily were more likely to be well educated, current smokers, and daily alcohol drinkers. Daily consumers who consumed more tea leaves per day tended to start drinking tea earlier and drink more cups of tea. The majority of CKB participants most commonly consumed green tea.

During a median of 10.1 years of follow-up and 4.5 million person years at risk in total, 12,130 participants experienced their first fracture hospitalization of any type, including 1376 cases of hip fracture. After adjustment for potential confounders, compared with participants who never drank tea during the past 12 months, daily tea consumption was associated with lower risk of any fracture (Table 2). Multivariable-adjusted HR (95% CI) was 0.88 (0.83, 0.93) for daily consumers. We did not observe a statistically significant linear trend in the risk of any fracture with the grams of tea leaves consumed in daily tea consumers (*p* for trend = 0.863). The corresponding HR (95% CI) for hip fracture was 0.84 (0.71, 1.00). Also, there was no linear trend for risk of hip fracture in daily consumers (*p* for trend = 0.148). Both associations were consistent between men and women (*p* for sex interaction: 0.960 for any fracture, 0.079 for hip fracture) (Table S1). The results were similar when daily consumers were further categorized by the cups of tea consumed (Table S2).

To test the robustness of our risk estimates, we performed sensitivity analyses by additionally adjusting for occupation, household income, consumption of calcium, iron, or zinc supplements in the whole cohort; or additionally adjusting for use of oral contraceptive in women; or excluding participants who were former tea consumers from the reference group (*n* = 3809); or excluding participants whose outcomes occurred during the first two years of follow-up (any fracture *n* = 1643, hip fracture *n* = 80). Also, open fractures of the clavicle (S42.0), proximal humerus (S42.2), and proximal or shaft tibia and fibula (S82.1, S82.2, and S82.4) were less likely related with osteoporosis [32]. Given that we could not distinguish open from closed fractures of these sites, we performed sensitivity analyses by further excluding them both as any fracture events. The associations were not substantially changed (data not shown).

**Figure 1:**
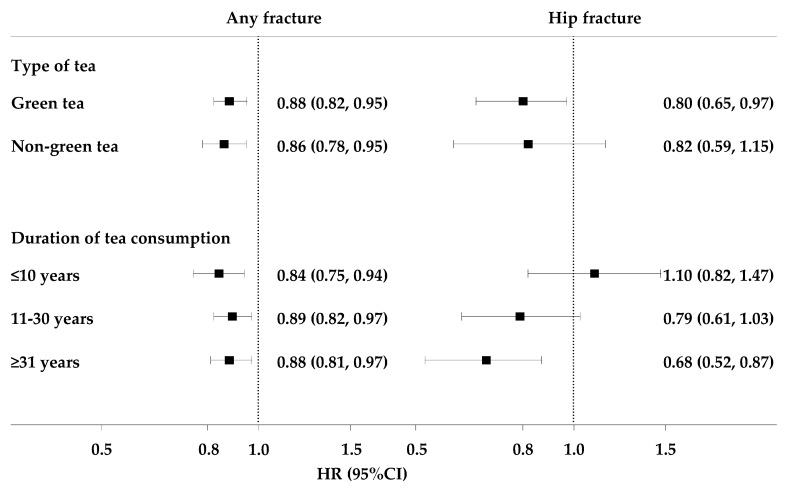
Subgroup analyses of associations between tea consumption and risk of fracture according to types of tea and duration of tea consumption in daily consumers. Hazard ratios are for comparison of daily tea consumers with participants who never consumed tea during the past 12 months. Risk estimates for participants who consumed tea less than daily are shown in Table S3. Solid squares represent point estimates, and horizontal lines represent 95% confidence intervals.

Figure 1 presents associations of tea consumption and fracture according to types of tea and duration of tea consumption in participants who drank tea daily. The risk estimates for any fracture seemed to be similar across different types of tea or duration of tea consumption. As for hip fracture, however, daily green tea consumers had a decreased risk (RR: 0.80; 95% CI: 0.65, 0.97). Strongest risk reduction for hip fracture was observed among daily consumers who had drunk tea for more than 30 years (RR: 0.68; 95% CI: 0.52, 0.87). Risk estimates for participants who consumed tea less than daily are shown in Table S3.

Further analyses were conducted according to prespecified baseline subgroups. Although statistically significant interaction with age was observed for incident any fracture (*p* values for interaction <0.001), the HRs were similar across different age subgroups. (Table S4). Associations between tea consumption and hip fracture were generally consistent across all subgroups (all *p* values for interaction >0.05/11).

## 4. Discussion

In this large prospective cohort study of Chinese adults, compared to participants who never drank tea during the past 12 months, daily tea consumers were associated with a 12% decreased risk of any fracture hospitalizations. Also, there was a suggestive association of a 16% lower risk of hip fracture hospitalizations among those who drank tea daily; such risk reduction became obvious among green tea consumers (18%) and prolonged tea consumers (32%). The associations were consistent in both men and women.

Existing prospective studies have yielded inconclusive results on the relationship between tea consumption and risk of fracture. Prospective studies conducted in Western women found no associations of tea consumption with the risks of hip fracture [18,19,20,24], forearm/wrist fracture [18,20], fracture other than hip and forearm/wrist [20], osteoporosis fracture [21], or any fracture [19]. However, these studies were primarily performed decades ago. Their confidence intervals of estimates tended to be wide due to small sample size and a limited number of outcomes, leading to less powerful results. The Singapore Chinese Health study with 16 years of follow-up and 2502 hip fracture cases also did not find associations between daily consumption of any tea and hip fracture (RR: 0.95; 95% CI: 0.85, 1.06) [22]. A cohort study of 1188 Australia women aged >75 years and 10-year follow-up showed that consumption of ≥3 cups of black tea per day was associated with a decreased risk of osteoporotic fracture that required hospitalization (RR: 0.70; 95% CI: 0.50, 0.96) compared with reference group of ≤1 cup per week [24]. Conversely, another study based on the Framingham cohort examined the association of caffeine consumption from both coffee and tea with hip fracture risk, with 135 cases occurred during 12 years of follow-up [23]. Its findings showed that consumption of ≥2.5 units of caffeine per day, an equivalent of 5 cups of tea, was associated with an increased risk of hip fracture. However, the results should be interpreted cautiously since researchers did not consider tea and coffee separately. 

In the current analysis of 10-year follow-up data of Chinese adults, we observed that daily tea consumption was associated with a reduction in risk of any fracture in both men and women. Despite the apparently smaller number of hip fracture cases, a reduced risk of hip fracture was also shown among participants with decades of tea consumption and those who were used to drinking green tea. The findings that tea consumption was associated with lower fracture risk are biologically reasonable based on previous experimental evidence. Tea polyphenols could enhance osteoblastogenesis, suppress osteoclastogenesis, increase bone formation, and inhibit bone resorption through antioxidant or anti-inflammatory pathways, which result in greater bone strength [9,10,11]. However, in the present study, the fracture risk associated with daily tea consumption did not follow any linear trend with the amount of tea leaves added, which may correspond to the amount of both polyphenols and caffeine in the tea. Adverse effects of increased caffeine on bone health may counterbalance the beneficial effect of tea polyphenols [8]. But at least we did not observe that stronger tea consumption, averaged about 9.5 g/day of tea leaves, increased the risk of fracture. Our findings also do not preclude the possibility that tea consumption exerts positive effects on attention and alertness throughout the day [33,34], reducing the risk of severe injury. Further studies are needed to verify our findings.

To the best of our knowledge, this is by far the largest prospective cohort study among a wide age range of Chinese adults investigating the association between habitual tea consumption and the risk of fracture. The strengths of our study included prospective study design, large study population, the inclusion of both men and women and a geographically spread Chinese population living in urban and rural areas, and available information on a broad range of covariates. We measured tea consumption in grams of tea leaves which may better reflect the intake of active ingredients and also collected detailed information on types of tea and duration of tea consumption. With the electronic linkage to local HI databases, we are able to obtain data on hospitalized fractures comprehensively, which could be difficult to be captured with the linkage to local disease and death registries or by face-to-face interviews.

Inevitably, there are some limitations in our study. First, there was measurement error in tea consumption since it was self-reported. However, misclassification in this prospective study should be non-differential and attenuate the associations to be null. Second, tea consumption was measured only once at baseline. However, tea consumers in our cohort usually had drunk tea for decades. Third, we could only ascertain fractures that required hospitalization. Underreporting of hospitalized fractures might exist, and other fractures that were not serious enough to lead to hospitalization were not captured in our study. Fourth, our analyses have adjusted for a range of potential confounders, but residual confounding may remain. For instance, coffee consumption, which was not collected at baseline survey, may be a confounder given that coffee may influence the risk of fracture [35]. However, the prevalence of coffee consumption is likely to be relatively low in our population. A re-survey involving about 5% of randomly chosen surviving participants was conducted during 2008, in which less than 2% of participants consumed coffee at least once a week. Thus the potential confounding from coffee consumption, if any, might be trivial. Fifth, we did not further analyze the effects of black tea, oolong tea or other types of tea on the risk of fracture separately since fewer participants consumed other types tea than green tea in our population. Sixth, we didn’t have data on bone mineral density at baseline survey, thus we couldn’t identify whether tea consumption reduced the risk of fracture through improving bone health.

## 5. Conclusions

In summary, this large prospective cohort of Chinese adults provided evidence that habitual tea consumption was associated with moderately decreased risk of any fracture hospitalizations in both men and women. Participants with decades of tea consumption and those who preferred green tea were also at lower risk of hip fracture. Given the observational nature of our study, causality cannot be established. Further randomized trials studies are needed to elucidate whether tea consumption has the potential to reduce the risk of fracture hospitalizations through improving bone health directly or improving attention and alertness, or if it is merely a marker of other dietary and lifestyle factors.

## Figures and Tables

**Table 1:** Baseline characteristics of 453,625 study participants according to tea consumption.

	Never	Less than Daily	Daily (Grams/Day)			
			0.1–2.0	2.1–3.0	3.1–5.0	>5.0
No. of participants, *n* (%)	159,367	175,569	45,835	20,505	25,373	26,976
	(35.1)	(38.7)	(10.1)	(4.5)	(5.6)	(5.9)
Age, year	52.5	49.8	52.8	52.6	52.4	51.4
Rural area, %	58.2	54.8	66.2	75.5	50.5	49.1
Married, %	89.1	91.3	92.2	92.5	93.3	93.8
Middle school and higher, %	40.6	51.6	54.9	57.4	59.3	59.8
Current smoker *, %						
Men	56.4	63.6	72.8	75.2	76.8	81.4
Women	2.3	2.7	4.3	5.3	4.8	6.9
Daily alcohol drinking, %						
Men	14.7	17.3	24.9	25.3	26.9	27.8
Women	0.6	0.9	2.3	2.9	2.6	3.9
Physical activity, MET h/day	21.2	21.9	21.0	21.4	21.2	21.5
Average weekly consumption †, day						
Red meat	3.44	3.73	3.98	3.75	4.04	4.24
Fresh vegetables	6.82	6.82	6.88	6.81	6.90	6.87
Fresh fruits	2.43	2.62	2.74	2.55	2.67	2.52
Dairy products	0.84	1.01	1.08	1.04	1.08	1.10
Body mass index, kg/m^2^	23.4	23.7	23.6	23.6	23.7	23.8
Waist-to-hip ratio	0.870	0.881	0.888	0.890	0.894	0.900
Diabetes, %	5.4	5.3	5.4	5.2	5.4	5.4
Hypertension, %	32.6	33.0	34.8	35.3	36.3	36.2
Postmenopausal (in women), %	50.4	49.7	49.4	49.1	48.9	48.8
Characteristics of daily tea consumer						
Age of starting tea consumption, year	-	-	28.4	27.8	26.6	25.0
Duration of tea consumption, year	-	-	23.9	24.5	25.8	27.4
Amount of tea consumption, gram	-	-	1.7	3.1	4.1	9.5
Amount of tea consumption, cup	-	-	3.3	4.2	4.6	6.6
Green tea consumer, %	-	-	86.0	85.7	86.1	85.8

Abbreviations: MET, metabolic equivalent of task. All variables were adjusted for age and survey areas, as appropriate. * Former smoker who had stopped smoking for illness was categorized into the current smoker. † Average weekly consumption of red meat, fresh vegetables, fresh fruits, and dairy products was calculated by assigning participants to the midpoint of their consumption category.

**Table 2:** HRs (95% CIs) for associations between tea consumption (in grams/day) and risk of fracture among 453,625 participants.

Endpoints	Never	Less than Daily	Daily (Grams/Day)					*p* for Trend *
			All	0.1–2.0	2.1–3.0	3.1–5.0	>5.0	
**Any fracture**								
No. of cases	4603	4502	3025	1237	471	621	696	
No. of PYs	1,568,372	1,737,307	1164,811	446,985	200,235	249,505	268,085	
Cases/PYs (/1000)	2.93	2.59	2.60	2.77	2.35	2.49	2.60	
Model 1	1.00	0.95 (0.91, 0.99)	0.89 (0.84, 0.94)	0.90 (0.84, 0.96)	0.85 (0.77, 0.94)	0.87 (0.80, 0.95)	0.91 (0.83, 0.99)	0.952
Model 2	1.00	0.95 (0.91, 1.00)	0.88 (0.83, 0.93)	0.90 (0.84, 0.96)	0.84 (0.76, 0.93)	0.86 (0.78, 0.94)	0.89 (0.81, 0.97)	0.807
Model 3	1.00	0.95 (0.91, 1.00)	0.88 (0.83, 0.93)	0.90 (0.84, 0.97)	0.84 (0.76, 0.94)	0.86 (0.79, 0.94)	0.89 (0.81, 0.97)	0.863
**Hip fracture**								
No. of cases	614	420	342	162	58	67	55	
No. of PYs	1,587,266	1,754,313	1,176,665	451,421	202,121	251,979	271,144	
Cases/PYs (/1000)	0.39	0.24	0.29	0.36	0.29	0.27	0.20	
Model 1	1.00	0.86 (0.75, 0.98)	0.82 (0.70, 0.97)	0.92 (0.76, 1.13)	0.73 (0.54, 0.98)	0.81 (0.62, 1.06)	0.70 (0.52, 0.93)	0.143
Model 2	1.00	0.87 (0.76, 0.99)	0.82 (0.70, 0.97)	0.92 (0.75, 1.12)	0.74 (0.55, 0.99)	0.80 (0.61, 1.05)	0.69 (0.51, 0.92)	0.119
Model 3	1.00	0.89 (0.77, 1.01)	0.84 (0.71, 1.00)	0.94 (0.77, 1.15)	0.76 (0.56, 1.02)	0.83 (0.63, 1.09)	0.71 (0.53, 0.96)	0.148

Abbreviations: HR, hazard ratio; CI, confidence interval; PYs, person years. Model 1 was adjusted for sex (men or women); model 2 additionally included level of education (no formal school, primary school, middle school, high school, college, or university or higher), marital status (married, widowed, divorced or separated, or never married), alcohol consumption (non-drinker, former weekly drinker, weekly drinker, daily drinking <15, 15–29, 30–59, or ≥60 g of pure alcohol), smoking status (never smoker, former smoker who had stopped smoking for reasons other than illness, current smoker, or former smoker who had stopped smoking for illness consuming 1–14, 15–24, or ≥25 cigarettes or equivalent per day), physical activity (MET h/day), frequencies of red meat, fruits, vegetables, and dairy products intake (daily, 4–6 days/week, 1–3 days/week, monthly, or rarely or never); model 3 additionally included BMI (kg/m^2^), waist-to-hip ratio, prevalent hypertension (presence or absence), and prevalent diabetes (presence or absence). * Tests for linear trend were only conducted in daily consumers by assigning the median value of tea consumption (in grams/day) to each of the categories as a continuous variable in regression models.

## Supplementary Materials

The following are available online at http://www.mdpi.com/2072-6643/10/11/1633/s1, Table S1: Sex-specific HRs (95% CIs) for associations between tea consumption (in grams/day) and risk of fracture among 453,625 participants, Table S2: HRs (95% CIs) for associations of tea consumption (in cups/day) and risk of fracture among 453,625 participants, Table S3: Subgroup analyses of associations between tea consumption and risk of fracture according to types of tea and duration of tea consumption, Table S4: Subgroup analyses of associations between tea consumption and risk of fracture according to potential baseline risk factors.
