# Supplementary material for: Habitual Tea Consumption and Risk of Fracture in 0.5 Million Chinese Adults: A Prospective Cohort Study
Source: Nutrients. 2018 Nov 2;10(11):1633. doi: 10.3390/nu10111633 (PMC6265708; doi:10.3390/nu10111633)
Supplement: Supplementary file 1 [file nutrients-10-01633-s001.pdf]

Supplementary Material

This file contains the following supplementary tables:

- **Table S1.** Sex-specific HRs (95% CIs) for associations between tea consumption (in grams /day) and risk of fracture among 453,625 participants.
- **Table S2.** HRs (95% CIs) for associations of tea consumption (in cups/day) and risk of fracture among 453,625 participants.
- **Table S3.** Subgroup analyses of associations between tea consumption and risk of fracture according to types of tea and duration of tea consumption.
- **Table S4.** Subgroup analyses of associations between tea consumption and risk of fracture according to potential baseline risk factors.

Table S1. Sex-specific HRs (95% CIs) for associations between tea consumption (in grams/day) and risk of fracture among 453,625 participants.

Endpoints	Never	Less than Daily	Daily (Grams/Day)					*P* for Trend *
			All	0.1–2.0	2.1–3.0	3.1–5.0	>5.0	
**Men**								
**Any fracture**								
No. of cases	684	1480	1902	643	269	443	547	
No. of PYs	337,136	696,809	728,494	233,880	110,286	174,624	209,704	
Cases/PYs (/1000)	2.03	2.12	2.61	2.75	2.44	2.54	2.61	
HRs (95% CIs)	1.00	0.90 (0.82, 0.99)	0.84 (0.76, 0.92)	0.87 (0.78, 0.98)	0.84 (0.73, 0.98)	0.82 (0.72, 0.92)	0.82 (0.73, 0.93)	0.405
**Hip fracture**								
No. of cases	104	118	194	85	27	46	36	
No. of PYs	339,933	702,958	736,317	236,260	111,425	176,453	212,179	
Cases/PYs (/1000)	0.31	0.17	0.26	0.36	0.24	0.26	0.17	
HRs (95% CIs)	1.00	0.65 (0.49, 0.86)	0.77 (0.59, 1.02)	0.95 (0.69, 1.31)	0.68 (0.43, 1.07)	0.79 (0.54, 1.14)	0.58 (0.39, 0.87)	0.046
**Women**								
**Any fracture**								
No. of cases	3919	3022	1123	594	202	178	149	
No. of PYs	1,231,235	1,040,498	436,318	213,106	89,949	74,881	58,382	
Cases/PYs (/1000)	3.18	2.90	2.57	2.79	2.25	2.38	2.55	
HRs (95% CIs)	1.00	0.96 (0.91, 1.01)	0.89 (0.82, 0.97)	0.90 (0.82, 0.99)	0.80 (0.68, 0.94)	0.89 (0.76, 1.04)	0.99 (0.84, 1.18)	0.143
**Hip fracture**								
No. of cases	510	302	148	77	31	21	19	
No. of PYs	1,247,333	1,051,355	440,347	215,161	90,696	75,526	58,965	
Cases/PYs (/1000)	0.41	0.29	0.34	0.36	0.34	0.28	0.32	
HRs (95% CIs)	1.00	0.98 (0.84, 1.14)	0.79 (0.63, 1.00)	0.81 (0.62, 1.08)	0.72 (0.48, 1.09)	0.74 (0.47, 1.17)	0.88 (0.54, 1.43)	0.544

Abbreviations: HR, hazard ratio; CI, confidence interval; PYs, person years. Multivariable model was adjusted for level of education (no formal school, primary school, middle school, high school, college, or university or higher), marital status (married, widowed, divorced or separated, or never married), alcohol consumption (non-drinker, former weekly drinker, weekly drinker, daily drinking <15, 15-29, 30-59, or ≥60 grams of pure alcohol), smoking status (never smoker, former smoker who

had stopped smoking for reasons other than illness, current smoker or former smoker who had stopped smoking for illness consuming 1–14, 15–24, or ≥25 cigarettes or equivalent per day), physical activity (MET h/day), frequencies of red meat, fruits, vegetables, and dairy products intake (daily, 4–6 days/week, 1–3 days/week, monthly, or rarely or never), menopausal status (premenopausal, perimenopausal, or postmenopausal; only in women analysis), BMI (kg/m^2), waist-to-hip ratio, prevalent hypertension (presence or absence), and prevalent diabetes (presence or absence). * Tests for linear trend were only conducted in daily consumers by assigning the median value of tea consumption (in grams/day) to each of the categories as a continuous variable in regression models.

Table S2. HRs (95% CIs) for associations of tea consumption (in cups/day) and risk of fracture among 453,625 participants.

Endpoints	Never	Less than Daily	Daily (Cups/Day)					*P* for Trend *
			All	1–2	3–4	5–6	≥7	
**Whole cohort**								
**Any fracture**								
No. of cases	4603	4502	3025	784	1065	640	536	
No. of PYs	1,568,372	1,737,307	1,164,811	289,621	416,715	258,830	199,644	
Cases/PYs (/1000)	2.93	2.59	2.60	2.71	2.56	2.47	2.68	
HRs (95% CIs)	1.00	0.95 (0.91, 1.00)	0.88 (0.83, 0.93)	0.93 (0.85, 1.01)	0.85 (0.79, 0.92)	0.90 (0.82, 0.98)	0.85 (0.77, 0.94)	0.294
**Hip fracture**								
No. of cases	614	420	342	111	121	67	43	
No. of PYs	1,587,266	1,754,313	1,176,665	292,416	420,992	261,312	201,945	
Cases/PYs (/1000)	0.39	0.24	0.29	0.38	0.29	0.26	0.21	
HRs (95% CIs)	1.00	0.89 (0.77, 1.01)	0.84 (0.71, 1.00)	0.98 (0.77, 1.23)	0.73 (0.59, 0.92)	0.88 (0.67, 1.15)	0.82 (0.59, 1.14)	0.673
**Men**								
**Any fracture**								
No. of cases	684	1480	1902	373	656	436	437	
No. of PYs	337,136	696,809	728,494	150,274	234,968	179,823	163,429	
Cases/PYs (/1000)	2.03	2.12	2.61	2.48	2.79	2.42	2.67	
HRs (95% CIs)	1.00	0.90 (0.82, 0.99)	0.84 (0.76, 0.92)	0.87 (0.76, 1.00)	0.88 (0.78, 0.98)	0.83 (0.73, 0.94)	0.77 (0.68, 0.88)	0.056
**Hip fracture**								
No. of cases	104	118	194	53	70	40	31	
No. of PYs	339,933	702,958	736,317	151,658	237,695	181,612	165,352	
Cases/PYs (/1000)	0.31	0.17	0.26	0.35	0.29	0.22	0.19	
HRs (95% CIs)	1.00	0.65 (0.49, 0.86)	0.77 (0.59, 1.02)	0.89 (0.61, 1.29)	0.78 (0.55, 1.09)	0.72 (0.48, 1.06)	0.70 (0.45, 1.08)	0.445
**Women**								
**Any fracture**								
No. of cases	3919	3022	1123	411	409	204	99	
No. of PYs	1,231,235	1,040,498	436,318	139,347	181,747	79,008	36,215	
Cases/PYs (/1000)	3.18	2.90	2.57	2.95	2.25	2.58	2.73	
HRs (95% CIs)	1.00	0.96 (0.91, 1.01)	0.89 (0.82, 0.97)	0.94 (0.84, 1.05)	0.76 (0.68, 0.86)	1.01 (0.87, 1.17)	1.06 (0.86, 1.31)	0.131
**Hip fracture**								
No. of cases	510	302	148	58	51	27	12	
No. of PYs	1,247,333	1,051,355	440,347	140,758	183,297	79,700	36,593	
Cases/PYs (/1000)	0.41	0.29	0.34	0.41	0.28	0.34	0.33	
HRs (95% CIs)	1.00	0.97 (0.83, 1.14)	0.79 (0.63, 1.00)	0.88 (0.64, 1.20)	0.58 (0.41, 0.81)	1.00 (0.66, 1.52)	1.09 (0.60, 1.98)	0.233

Abbreviations: HR, hazard ratio; CI, confidence interval; PYs, person years. Multivariable model was adjusted for sex (men or women; only in whole cohort analysis), level of education (no formal school, primary school, middle school, high school, college, or university or higher), marital status (married, widowed, divorced or separated, or never married), alcohol consumption (non-drinker, former weekly drinker, weekly drinker, daily drinking <15, 15–29, 30–59, or ≥60 grams of pure alcohol), smoking status

(never smoker, former smoker who had stopped smoking for reasons other than illness, current smoker or former smoker who had stopped smoking for illness consuming 1–14, 15–24, or ≥25 cigarettes or equivalent per day), physical activity (MET h/day), frequencies of red meat, fruits, vegetables, and dairy products intake (daily, 4–6 days/week, 1–3 days/week, monthly, or rarely or never), menopausal status (premenopausal, perimenopausal, or postmenopausal; only in women analysis), BMI (kg/m^2), waist-to-hip ratio, prevalent hypertension (presence or absence), and prevalent diabetes (presence or absence). * Tests for linear trend were only conducted in daily consumers by assigning the median value of tea consumption (in cups/day) to each of the categories as a continuous variable in regression models.

Table S3 Subgroup analyses of associations between tea consumption and risk of fracture according to types of tea and duration of tea consumption.

Subgroups	Never			Less than Daily *			Daily		
	No. of Cases	Cases/PYs (/1000)	HR	No. of Cases	Cases/PYs (/1000)	HRs (95% CIs)	No. of Cases	Cases/PYs (/1000)	HRs (95% CIs)
**Any fracture**	4603	2.93	1.00						
**Type of tea**									
Green tea				574	2.12	0.90 (0.82, 1.00)	2290	2.29	0.88 (0.82, 0.95)
Non-green tea				207	3.61	0.95 (0.82, 1.09)	735	4.50	0.86 (0.78, 0.95)
**Duration of tea consumption**									
≤10 years				212	1.96	0.94 (0.81, 1.09)	389	2.14	0.84 (0.75, 0.94)
11-30 years				358	2.20	0.91 (0.81, 1.03)	1328	2.28	0.89 (0.82, 0.97)
≥31 years				211	3.74	0.93 (0.80, 1.08)	1308	3.26	0.88 (0.81, 0.97)
**Hip fracture**	614	0.39	1.00						
**Type of tea**									
Green tea				50	0.18	0.70 (0.51, 0.96)	290	0.29	0.80 (0.65, 0.97)
Non-green tea				11	0.19	0.59 (0.32, 1.10)	52	0.31	0.82 (0.59, 1.15)
**Duration of tea consumption**									
≤10 years				8	0.07	0.36 (0.18, 0.74)	58	0.32	1.10 (0.82, 1.47)
11-30 years				26	0.16	0.79 (0.52, 1.20)	106	0.18	0.79 (0.61, 1.03)
≥31 years				27	0.47	0.79 (0.52, 1.21)	178	0.44	0.68 (0.52, 0.87)

Abbreviations: HR, hazard ratio; CI, confidence interval; PYs, person years. Multivariable model was adjusted for sex (men or women), level of education (no formal school, primary school, middle school, high school, college, or university or higher), marital status (married, widowed, divorced or separated, or never married), alcohol consumption (non-drinker, former weekly drinker, weekly drinker, daily drinking <15, 15–29, 30–59, or ≥60 grams of pure alcohol), smoking status (never smoker, former smoker who had stopped smoking for reasons other than illness, current smoker or former smoker who had stopped smoking for illness consuming 1–14, 15–24, or ≥25 cigarettes or equivalent per day), physical activity (MET h/day), frequencies of red meat, fruits, vegetables, and dairy products intake (daily, 4–6 days/week, 1–3 days/week, monthly, or rarely or never), BMI (kg/m^2), waist-to-hip ratio, prevalent hypertension (presence or absence), and prevalent diabetes (presence or absence). * Excluded 142,576 participants who consumed tea 'only occasionally, only at certain seasons, or monthly but less than weekly' and were not asked to report the commonly consumed tea type or years of tea consumption.

Table S4. Subgroup analyses of associations between tea consumption and risk of fracture according to potential baseline risk factors.

Subgroups	Never		Less than daily		Daily		$P_{interaction}$ †
	No. of Cases	HR	No. of Cases	HR (95%CI)	No. of Cases	HR (95%CI)	
**Any fracture**							
**Age at baseline (years)**							
<50	1007	1.00	1487	1.00 (0.91, 1.09)	989	0.90 (0.81, 1.00)	<0.001
≥50	3596	1.00	3015	0.96 (0.91, 1.01)	2036	0.89 (0.84, 0.96)	
**Region**							
Rural	3067	1.00	3269	0.95 (0.90, 1.01)	2267	0.85 (0.79, 0.91)	0.258
Urban	1536	1.00	1233	0.95 (0.87, 1.03)	758	0.96 (0.86, 1.06)	
**Alcohol consumption (g/day)**							
<30	4510	1.00	4167	0.95 (0.91, 1.00)	2434	0.88 (0.82, 0.93)	0.218
≥30	93	1.00	335	1.07 (0.85, 1.36)	591	1.01 (0.80, 1.27)	
**Smoking status**							
Not current	4080	1.00	3285	0.95 (0.91, 1.00)	1425	0.87 (0.81, 0.94)	0.765
Current	523	1.00	1217	0.93 (0.84, 1.04)	1600	0.88 (0.79, 0.98)	
**Physical activity (MET h/day)**							
<12.29	1439	1.00	1166	0.93 (0.86, 1.01)	919	0.84 (0.76, 0.94)	0.031
12.29 to <25.30	1381	1.00	1632	0.96 (0.89, 1.04)	1030	0.90 (0.82, 1.00)	
≥25.30	1783	1.00	1704	0.95 (0.88, 1.02)	1076	0.87 (0.79, 0.95)	
**BMI (kg/m^2)**							
<18.5	2859	1.00	2621	0.92 (0.87, 0.98)	1921	0.87 (0.81, 0.94)	0.116
18.5 to 24.0	1353	1.00	1464	1.01 (0.93, 1.10)	842	0.89 (0.80, 0.98)	
≥24.0	391	1.00	417	0.94 (0.81, 1.09)	262	0.87 (0.72, 1.05)	
**Central obesity***							
No	3014	1.00	2855	0.94 (0.89, 0.99)	2126	0.89 (0.83, 0.95)	0.060
Yes	1589	1.00	1647	0.98 (0.91, 1.05)	899	0.86 (0.78, 0.95)	
**Hypertension**							
No	2556	1.00	2901	0.97 (0.91, 1.02)	1838	0.91 (0.85, 0.98)	0.032
Yes	2047	1.00	1601	0.94 (0.88, 1.01)	1187	0.84 (0.77, 0.92)	
**Diabetes**							
No	4223	1.00	4167	0.94 (0.90, 0.99)	2825	0.87 (0.82, 0.92)	0.473
Yes	380	1.00	335	1.11 (0.95, 1.30)	200	1.00 (0.82, 1.23)	
**Postmenopausal (in women)**							
No	914	1.00	914	0.91 (0.82, 1.01)	348	0.81 (0.70, 0.95)	0.716
Yes	3005	1.00	2106	0.98 (0.92, 1.04)	775	0.92 (0.84, 1.02)	
**Hip fracture**							
**Age at baseline (years)**							
<50	40	1.00	66	1.03 (0.67, 1.60)	42	0.78 (0.46, 1.32)	0.377

Subgroups	Never		Less than daily		Daily		$P_{interaction}$ †
	No. of Cases	HR	No. of Cases	HR (95%CI)	No. of Cases	HR (95%CI)	
≥50	574	1.00	354	0.88 (0.76, 1.02)	300	0.87 (0.73, 1.03)	
**Region**							
Rural	298	1.00	228	0.84 (0.69, 1.02)	218	0.73 (0.58, 0.92)	0.158
Urban	316	1.00	192	0.92 (0.76, 1.11)	124	1.00 (0.78, 1.28)	
**Alcohol consumption (g/day)**							
<30	606	1.00	401	0.90 (0.78, 1.03)	300	0.84 (0.70, 1.00)	0.540
≥30	8	1.00	19	0.87 (0.36, 2.13)	42	0.99 (0.43, 2.29)	
**Smoking status**							
Not current	534	1.00	325	0.92 (0.79, 1.07)	187	0.84 (0.68, 1.04)	0.379
Current	80	1.00	95	0.77 (0.56, 1.07)	155	0.84 (0.61, 1.16)	
**Physical activity (MET h/day)**							
<12.29	337	1.00	202	0.83 (0.69, 1.01)	177	0.72 (0.57, 0.91)	0.704
12.29 to <25.30	164	1.00	141	1.05 (0.83, 1.35)	101	1.07 (0.79, 1.46)	
≥25.30	113	1.00	77	0.79 (0.58, 1.08)	64	0.90 (0.61, 1.32)	
**BMI (kg/m²)**							
<18.5	422	1.00	262	0.82 (0.70, 0.97)	239	0.81 (0.66, 1.00)	0.595
18.5 to 24.0	151	1.00	124	0.99 (0.77, 1.29)	79	0.85 (0.61, 1.19)	
≥24.0	41	1.00	34	0.96 (0.58, 1.58)	24	0.84 (0.45, 1.56)	
**Central obesity***							
No	381	1.00	259	0.90 (0.76, 1.07)	233	0.89 (0.72, 1.09)	0.866
Yes	233	1.00	161	0.87 (0.70, 1.08)	109	0.76 (0.57, 1.01)	
**Hypertension**							
No	287	1.00	226	0.89 (0.74, 1.08)	171	0.87 (0.68, 1.10)	0.930
Yes	327	1.00	194	0.88 (0.72, 1.06)	171	0.82 (0.65, 1.04)	
**Diabetes**							
No	524	1.00	368	0.90 (0.78, 1.04)	300	0.84 (0.70, 1.00)	0.768
Yes	90	1.00	52	0.81 (0.56, 1.17)	42	0.92 (0.58, 1.44)	
**Postmenopausal (in women)**							
No	43	1.00	43	0.88 (0.55, 1.41)	26	0.68 (0.36, 1.29)	0.856
Yes	467	1.00	259	0.99 (0.84, 1.17)	122	0.81 (0.63, 1.05)	

Abbreviations: HR, hazard ratio; CI, confidence interval; MET, metabolic equivalent of task. Except for the baseline stratifying variable, multivariable models were adjusted for sex (men or women), level of education (no formal school, primary school, middle school, high school, college, or university or higher), marital status (married, widowed, divorced or separated, or never married), alcohol consumption (non-drinker, former weekly drinker, weekly drinker, daily drinking <15, 15–29, 30–59, or ≥60 grams of pure alcohol), smoking status (never smoker, former smoker who had stopped smoking for reasons other than illness, current smoker or former smoker who had stopped smoking for illness consuming 1–14, 15–24, or ≥25 cigarettes or equivalent per day), physical activity (MET h/day), frequencies of red meat, fruits, vegetables, and dairy products intake (daily, 4–6 days/week, 1–3 days/week, monthly, or rarely or never), BMI (kg/m²), waist-to-hip ratio, prevalent hypertension (presence or absence), and prevalent diabetes (presence or absence). * Central obesity was defined as waist-to-hip ratio ≥0.95 in men or ≥0.90 in women. † The tests for

interaction were performed using likelihood ratio tests, which involved comparing models with and without cross-product terms between the baseline stratifying variable and tea consumption as an ordinal variable.
